# Whole-genome sequencing

**DOI:** 10.1136/practneurol-2020-002561

**Published:** 2021-05-10

**Authors:** Huw R Morris, Henry Houlden, James Polke

**Affiliations:** 1 Department of Clinical and Movement Neuroscience, UCL Queen Square Institute of Neurology, London, UK; 2 Department of Molecular Neuroscience, UCL Queen Square Institute of Neurology, London, UK; 3 Neurogenetics, University College London Hospitals NHS Foundation Trust National Hospital for Neurology and Neurosurgery, London, UK

**Keywords:** neurogenetics

## Abstract

The costs of whole-genome sequencing have rapidly decreased, and it is being increasingly deployed in large-scale clinical research projects and introduced into routine clinical care. This will lead to rapid diagnoses for patients with genetic disease but also introduces uncertainty because of the diversity of human genomes and the potential difficulties in annotating new genetic variants for individual patients and families. Here we outline the steps in organising whole-genome sequencing for patients in the neurology clinic and emphasise that close liaison between the clinician and the laboratory is essential.

## Introduction

Making a genetic diagnosis is important. For people with familial or early-onset disease, a molecular genetic diagnosis may minimise the need for further investigation, allow accurate counselling on inheritance and familial risk and increasingly define eligibility for therapeutic trials such as antisense oligonucleotide therapy directed towards the underlying genetic cause. Most neurologists are comfortable with requesting and interpreting single-gene tests for neurological conditions, for example, the Huntington’s disease test for someone affected by chorea. However, many conditions have an increasing number of potential causative genes. For hereditary neuropathy, at least 75 genes may cause the Charcot-Marie-Tooth syndrome, and pathogenic mutations in many other genes may cause syndromes that include neuropathy.^1^

The advent of high-throughput parallel DNA sequencing means that these potential genetic causes can be rapidly evaluated, and the patient’s DNA sequence can be determined accurately and reasonably cheaply when compared with tests like magnetic resonance scanning. The field has moved from Sanger sequencing and fragment analysis (1–10 genes) to resequencing panels (a battery of 10–100 genes), whole-exome sequencing (~20 000 protein-coding genes) and now whole-genome sequencing (including coding and non-coding genes, DNA repeat expansions, regulatory regions and information on structural variation).

The UK has positioned itself to be at the forefront of the integration of genomics in medicine, stimulated by the support for the 100,000 Genomes Project and the implementation of a process for whole-genome sequencing delivered by the NHS England Genomic Medicine Service.^2^ However, the increasing amounts of data generate increasing challenges in interpretation and generation of clear answers for patients and clinicians. Here, we outline some key considerations in providing genome sequencing for people with neurological disease. All neurologists need to become familiar with these issues as this becomes a mainstream technology; close working between the clinicians, specialist clinics and diagnostic labs will be crucial.

The first draft human genome sequence was released in 2001, and the 2020 release of the human Genome Aggregation Database (gnomAD) contains variant data derived from whole genomes from 15 708 people and whole exomes from 125 748 people. One of the revelations of the progress in human genome sequencing is the amount of diversity between human genomes. The average genome contains ~8500 novel single-nucleotide variants.^3^ With respect to Mendelian disease, individual pathogenic mutations at a population level are always rare. However, across the ~20 000 human genes, each individual can be estimated to carry 16 rare putative loss of function mutations and 10 rare structural variants, each of which will lead to loss of one functional allele.^4 5^ Carrier status, that is, carrying one loss of function allele for a recessive disease gene, usually has no deleterious health implications.

Whole-genome sequencing has a high diagnostic yield in people who are very likely to have single-gene diseases. These patients can be defined by childhood, juvenile and early-onset diseases (likely to be due to de novo or autosomal recessive mutations, if genetic) and familial diseases where there are many family members affected by disease. However, in neurology, many clinically identical diseases can be polygenic (likely due to many risk genes of low penetrance, with environmental factors) or due to a single-gene mutation. Similarly, some diseases such as early-onset severe epilepsy may be environmental, relating to perinatal anoxic–ischaemic brain damage, or due to genetic changes, in particular de novo mutations. The situation is further complicated by reduced penetrance for some disease genes and opaque family histories, for example, in kindreds with frontotemporal dementia where some individuals with the condition have been diagnosed as having a psychiatric illness. It is important to make an estimate of the a priori likelihood of a genetic diagnosis. Gathering as much information as possible about the family history is a crucial part of the neurogenetics clinical evaluation. Conversely, neurodegenerative diseases are common in later life and there are published outlines to help to establish the likelihood of a single-gene mutation, for example, the Goldman score in frontotemporal dementia.^6^

## Who to test

Before arranging whole-genome sequencing, it is worth considering who is most likely to benefit. The 100,000 Genomes Project is a UK NHS-led research project in which approximately 78 000 individuals in families with rare disease and 22 000 patients with cancer had blood (germline) whole-genome sequencing (together with cancer sequencing to explore the occurrence of somatic mutations). This was an unprecedented opportunity to explore the implementation of whole-genome sequencing in healthcare and to optimise patient selection, consent, interpretation and feedback. This has been followed by the development of the NHS England Genomic Medicine services plan and the setting up of seven genomics lab hubs across England (https://www.england.nhs.uk/genomics/nhs-genomic-med-service/).

Neurology (including paediatric diseases and intellectual disability) was the largest single medical specialty contributing cases to the 100,000 Genomes Project. The overall diagnostic rate from the project was around 25% (that is a genetic diagnosis was made in 25% of families), and the diagnostic yield was greater when trios and larger families were available for analysis, so where possible, collecting samples from multiple family members is very helpful. For the families most likely to have a single-gene cause, around 40% of cases had identified pathogenic mutations. In the clinic, younger people, for example, the son or daughter of a patient with familial neurodegenerative disease, frequently volunteer to be tested to help with their parent’s analysis. Usually, this is not helpful as those below the predicted age of onset may be asymptomatic mutation carriers, and so analysing their genome will not help to narrow down the pathogenic mutation. Conversely, analysing the genomes of the parents of a child with childhood-onset illness is extremely helpful as loss of function variants in the healthy unaffected parents can effectively be excluded as causative mutations. In some families, younger at-risk individuals may volunteer for genetic testing, motivated by a desire to know their genetic status. Predictive testing (of at-risk unaffected individuals) should only be carried out with appropriate genetic counselling, on inheritance, recurrence risk and penetrance, and predictive testing protocols in a genetics clinic.

## How to take consent

Standard consent for any genetic test should include an explanation of the implication of a positive result, that is, the potential identification of risk to other family members as well as potential implications for the patient, including prognosis, screening, therapy and research. Whole-genome sequencing involves several additional considerations, related to large-scale analysis that are not necessarily relevant to single-gene testing. The NHS England genomics medicine service has issued a standard template for consent, which includes documentation of a consent discussion on whole-genome sequencing with the family. The standard consent discussion now includes (1) the comparison of results with results from other similar families, (2) the possibility of ambiguous or uncertain results, (3) long-term storage of genomic data and (4) the possibility of ‘unexpected’ results. It is important to emphasise that, currently, about half of whole-genome sequencing tests will not lead to a definitive result and that the test results may take up to a year to become available.

## What to test

Although it is cost effective to sequence all the genes in the genome in one test, when it comes to analysis, a restricted gene list is evaluated (gene panel or ‘virtual’ gene panel, relevant to a disease group such as hereditary spastic paraparesis or ataxia). It is important that irrelevant genes are not tested, and because of variation in the human genome, the larger the number of genes tested, the higher the chance of a false-positive result. The gene lists for each phenotype in the 100,000 Genomes Project have been collated with public facing software (PanelApp, https://panelapp.genomicsengland.co.uk/), which enables anyone in the clinical and research community to comment on the genes included in a gene panel with a traffic light system. Thus, green is for high level of evidence for gene–disease association—the gene can be used in genome interpretation; amber—pause–moderate evidence for gene–disease association—should not be used for genome interpretation; red—stop—the gene should not be used for genome interpretation. The PanelApp system and the traffic light approach encompass the dynamic nature of gene–phenotype association with new pathogenic gene mutations being described and sometimes refuted on a monthly basis. In practical terms, it is important that the clinician specifies the phenotype that will be used by the laboratory, and (for some specific phenotypes) checks that the relevant gene is included in the panel. Further clinical input on phenotype and results is needed when the results are generated. It is important neurologists are aware that not all of the genes reported in the literature as being a ‘new’ gene for a specific condition will be confirmed by subsequent reports and will reach the standard needed for confident genetic diagnosis and counselling, and may not be included in the gene panel. The clinician is crucial in accurately defining the phenotype and the correct panel to be interpreted in conjunction with the genetics laboratory.

## How to interpret the diagnostic results

Following the selection of the gene panels and the extraction of variants as compared with the reference genome, for an individual from those gene panels, comes the evaluation of whether the variants are likely to be pathogenic. The large number of rare variants in each genome presents potential challenges in interpretation of the results, and this is the area with which most neurologists will be least familiar. Clearly, the larger the number of genes investigated, the higher the chance of false positives (an irrelevant rare variant with no implications for diagnosis or genetic counselling).

Pathogenic gene variants are usually rare in the general population, and this is an important factor in the initial triage of the variant. For example, the common *DYT1/TOR1A* deletion mutation c.907_909delGAG, leading to a deletion of a single glutamic acid at codon 303 (which has reduced penetrance), causes autosomal dominant primary dystonia, with reduced penetrance. The frequency of the *DYT1* GAG deletion can be estimated to be 0.02% (gnomAD v3) with 30 pathogenic alleles seen in ~140 000 genomes/exomes. This is a relatively common disease allele likely explained in part by the reduced (30%) penetrance. Very often dominant pathogenic variants are absent from gnomAD and dominant alleles with a frequency of >0.02% are unlikely to be pathogenic. There is a useful framework that considers the genetic disease prevalence and genetic architecture to find the maximum credible allele frequency, that is, the highest frequency expected in the general population.^7^ For example, the maximum credible allele count in gnomAD for dominant CMT is 3/280 000 alleles.^8^

Further annotation of the variant can be made based on the type of variant, and there are several tools and approaches to interpreting rare genetic variants. A detailed description of the framework for classifying variants is outside the scope of this paper but has been published by the American College of Medical Genetics (ACMG).^9^ Sequence variants are conventionally defined as pathogenic, likely pathogenic, uncertain significance, likely benign or benign (ACMG classes 5, 4, 3, 2 and 1, respectively). The criteria include segregation, that is, the previous occurrence of the mutation in individuals with the same phenotype in previous reports and within the family under investigation. Analysis of parental DNA is important in childhood onset disease, and analysis of older people can help in the interpretation of late-onset disease. Parental DNA analysis helps in defining de novo mutations and in defining ‘phase’ in autosomal recessive disease. Phase in a diagnostic genetics context refers to whether two deleterious recessive disease mutations in a patient lie on the paternal and maternal chromosomes or whether they are on a single chromosome, in which case the patient effectively has one normal copy of the gene and is unlikely to have an autosomal recessive disease mechanism. The simplest way to define this is analysis of maternal/paternal DNA. Frequently, in adult-onset recessive disease, parental DNA is not available and a biallelic mechanism is inferred by the distance between the mutations (lying in different exons) and the absence of previously reported coinheritance. Sources for previous disease associations include the ClinVar database and Human Gene Mutation Database,^10 11^ which provide a source for collation of reports from disease-mutation reports from clinical diagnostic laboratories, research laboratories and expert panels.

If the mutation is novel, it may be important to predict the functional effects of the rare variant in a disease gene. Putative loss-of-function mutations are usually reasonably clear-cut and can be used in defining individuals likely to harbour two loss-of-function alleles in autosomal recessive disease, or a single loss-of-function allele in some de novo mutation conditions where the gene is sensitive to haploinsufficiency. Coding, non-synonymous single-nucleotide variants that lead to a change in amino acid sequence, that have not previously been shown to segregate with disease, are particularly challenging. Factors to consider include conservation (whether the variant is conserved in evolutionary terms across species), local constraint (tolerance of the gene or region to non-synonymous variation) and the prediction of the effect of the variant on protein function, based on in silico algorithms. It is likely that in the future we will use high-throughput functional (cell-based) assays to determine at a functional level whether the variants share a common pathogenic mechanism with well-established disease mutations. Further investigation can help, for example, in looking at muscle biochemistry in patients carrying mitochondrial disease-causing variants, or in specific features on brain imaging. For example, pathogenic *LRRK2* mutations increase LRRK2 kinase activity, so it would be possible to evaluate new mutations to establish whether they share a common functional profile with the known *LRRK2* G2019S mutation.

A potential pitfall is the interpretation of ACMG grade 3 variants of unknown significance in plausible disease genes. When investigating next-generation sequencing, and particularly genome sequencing data, several grade 3 variants of unknown significance will be identified; it is important to interpret these carefully and to avoid suggesting a variant of unknown significance could be pathogenic if it is found in a candidate gene without further supportive evidence. To try and strengthen or repudiate a ACMG grade 3 variant of unknown significance, clinicians should seek further evidence for segregation of the variant with the disease either within or across families/individuals. Extra family members should be analysed, with unaffected and ideally affected members for segregation. The multidisciplinary team should search to find if the variant has been previously published or if other laboratories in the UK genetic testing network have identified the variant in affected family members, to provide further evidence for segregation and pathogenicity.

A ‘negative’ whole-genome sequencing result does not mean that the condition is not genetic, or that there is no recurrence risk to relatives. It is likely that there are many genes for neurological disease that have not yet been identified, and this may relate in part to non-coding DNA variation, including repeat expansions and structural rearrangements.

In understanding genetic variants, there should be a system for a regular liaison between neurology clinicians and laboratory staff or clinical genetics teams. We recommend regular multidisciplinary team meetings that may focus on a particular group of clinicians, such as neuromuscular or dementia or a general neurology/diagnostic laboratory group.

From the neurologists’ standpoint it is worthwhile emphasising that the clinician’s role is essential in ensuring that the phenotype has been correctly defined, evaluating further family members for segregation, and in helping the diagnostics laboratory to evaluate the likely role of pathogenic mutations identified in genome sequencing—the genome cannot be interpreted without the clinical data. Specialist genetics/neurogenetics clinics may be particularly important in the diagnostic phase in characterising complex phenotypes in multiple family members and in liaising with the genomics laboratory in interpreting genomic variants. All clinicians talking on the organisation of whole-genome sequencing should be prepared to participate in a multidisciplinary team meeting with the genomic testing laboratory.

## How to deal with unexpected findings

Whole-genome sequencing may identify variants unrelated to the primary condition under investigation, which may be important for the risk of future disease. This might, for example, relate to increases in the risk of developing Alzheimer’s disease or Parkinson’s disease, or the future risk of bowel or breast cancer. These potentially medically important findings, unrelated to the primary condition, have been variously referred to as additional, incidental or unexpected findings. The clinical management of patients carrying these variants has been considered by consensus panels of the ACMG. The American guidelines recommend prospectively looking in 59 ‘actionable’ genes for any patient undergoing whole-exome/whole-genome sequencing, regardless of the indication, with the patient having the ability to opt out.^12^ If the mutation is in a gene for which there is a known beneficial healthcare intervention, for example, breast cancer surveillance and counselling in a family carrying a BRCA1 mutation, or cardiology review in patients carrying mutations in long QT syndrome genes (ie, medically actionable), and if the patient has consented to receive these results, they would be fed back to them and the appropriate steps would be taken. The ACMG recommendations have been widely debated as they have developed,^13^ and there is discussion over the introduction of equivalent UK recommendations.^14^ Patients having whole-genome sequencing in the Genomic Medicine Service in England will not have additional findings looked for at first, and the future approach will be guided by the findings from the 100,000 Genomes Project.

The list of potentially actionable genes will probably grow and there will be increasing numbers of variants that change healthcare, for example, in predicting medication adverse effects and in defining eligibility for preventative screening programmes. Importantly, for the clinician arranging the tests, at the consent stage, the patient needs to confirm that they understand that the test may reveal unexpected results that are not related to their condition. In our experience, almost all patients in clinical or research programmes agree to this future feedback when offered. Importantly, the results of risk factor gene analysis, for variants that confer a relatively low risk (eg, ApoEe4) or genes for which there is currently no therapeutic intervention (eg, Huntington’s disease gene pathogenic expansion), are not fed back to patients as additional findings. Conversely, it is important for the patient and the clinician not to assume that a test, for example, for ataxia, will cover all other genetic conditions. If the patient has a family history of, for example, breast or colon cancer, they should be referred to the appropriate breast or colon cancer genetics/oncology screening service, regardless of any genetic tests that they may be having for other conditions.

## How to give the results

Following a positive genetic test, the patient needs genetic counselling and advice. Practice may vary in different clinics and different healthcare systems, but broadly, it is reasonable for the neurologist to explain the type of inheritance pattern, prognosis, availability of clinical interventions, research and patient support groups based on the primary genetic diagnosis. The GeneReviews website is a useful resource outlining the clinical and counselling aspects of genetic diseases. These issues may be best discussed in a specialist genetics/neurogenetics clinic. Certainly, when it comes to evaluation and counselling of the wider family and consideration of predictive/antenatal testing, this can only be carried out in specialist genetics/neurogenetics clinic setting, with appropriate counselling and support, and onward referral to a specialist clinic should be considered for all patients.

## Conclusions

The advent of whole-genome sequencing will immediately speed up the provision of accurate genetic diagnoses for our patients. The ability for whole-genome sequence data to be held centrally effectively as part of the medical record means that patient genomes can be re-evaluated at several points through life. For example, a genome generated on unaffected parents in their 20s may be evaluated for pharmacogenetic predictors of response to antihypertensive therapy in their 50s. Neurologists will need to become familiar with the processes both for consent and counselling for whole-genome sequencing. Our increasing understanding of the interplay between the genome, disease risk and response to treatment means that this should dramatically improve the outlook for patients with neurological disease, and an increasing amount of genomic information will become medically important.

**Key points**

- The human genome is diverse, with each individual carrying multiple rare single-nucleotide variants and structural variants, meaning that it may be difficult to ascribe a variant to a disease phenotype.
- Some disease phenotypes have many potential causative genes increasing the chance of false-positive results.
- If possible, recruit multiple family members, including older unaffected people for analysis.
- Liaison between the referring clinical and the diagnostic testing laboratory is very important in helping to interpret the results.
- Genome data can be reinterrogated in the light of future clinical phenotypes and may also contain incidental/additional findings that may be relevant for disease management or prevention unrelated to the original disease.

**Further reading**

- Keogh, M. J., Daud, D., & Chinnery, P. F. (2013). Exome sequencing: how to understand it. Practical Neurology, 13(6), 399–407.
- NHS Genomic Medicine Service: https://www.england.nhs.uk/genomics/nhs-genomic-med-service/
- 100,000 Genomes Project: https://www.genomicsengland.co.uk/about-genomics-england/the-100000-genomes-project/
